# Editorial: Safety of corticosteroids in respiratory medicine

**DOI:** 10.3389/fdsfr.2025.1656299

**Published:** 2025-07-28

**Authors:** Semra Bilaçeroğlu

**Affiliations:** Izmir Faculty of Medicine, University of Health Sciences, Izmir, Türkiye

**Keywords:** corticosteroids, safety, adverse effect, efficacy, respiratory diseases

The history of corticosteroids in medicine started in 1930 with the studies showing that adrenocortical samples from animals could reverse adrenal failure when given to humans (Rowntree et al., 1931). By 1940, it had been recognized that there were two groups of corticosteroids: those leading to sodium-fluid retention and those counteracting inflammation and shock (Kendall, 1941; Benedek, 2011). The treatment of a rheumatoid arthritis patient with cortisone in 1948 was the first step of their use in medical practice (Hench et al., 1949). Oral and intraarticular cortisone/hydrocortisone treatment started in 1950–1951 (Benedek, 2011; Freyberg et al., 1950). After several studies until 1952, six synthetic corticosteroids were introduced for systemic anti-inflammatory treatment between 1954–1958 (Benedek, 2011; Bunim et al., 1955). Their complete side effect profile and how to withdraw them while minimizing adrenal insufficiency were described in-detail by 1960 (Benedek, 2011; Katz and Katz, 1988).

Systemic corticosteroids are effective, cheap and easily accessible. In respiratory medicine, they are commonly used for treating severe viral and bacterial community-acquired pneumonia (CAP), acute respiratory distress syndrome (ARDS), septic shock, interstitial lung disease (ILD), chronic obstructive pulmonary disease (COPD) and asthma. The therapeutic effect of corticosteroids is mostly through their anti-inflammatory or immunosuppressive features (Melani et al., 2023; Janahi et al., 2018; Chang et al., 2022; Tiwari et al., 2024).

Excluding influenza infections, in severe viral CAP with an inflammatory cytokine storm, corticosteroids can reduce upregulated inflammatory responses. In severe bacterial CAP (excluding tuberculosis), symptom resolution and time to clinical stability may be accelerated by corticosteroids through suppression of high inflammatory response (Melani et al., 2023; Janahi et al., 2018). In ARDS, corticosteroids reduce inflammation due to diffuse lung injury, and consequently in-hospital mortality. In sepsis/septic shock, they suppress life-threatening host response to an infection that can lead to multiorgan failure (Melani et al., 2023; Janahi et al., 2018; Chang et al., 2022). In ILDs (excluding idiopathic pulmonary fibrosis-IPF), corticosteroid therapy for rapid progression and/or acute exacerbations may prevent decline in lung function and quality of life (QoL) (Melani et al., 2023; Janahi et al., 2018). In COPD, acute exacerbations are treated with inhaled/systemic corticosteroids to shorten recovery time, and to reduce early relapses and treatment failure (Melani et al., 2023; Janahi et al., 2018; Tiwari et al., 2024). In asthma, inhaled/systemic corticosteroids are useful in the treatment of acute exacerbations as well as long-term maintenance therapy for controlling asthma. By blocking the NFκB pathway besides reducing phospholipase A2 expression and leukotrienes, they further bronchodilation. Their immunosuppresive effect reduces IgE, and their long-term anti-inflammatory activity can impede airway remodelling (Melani et al., 2023; Janahi et al., 2018).

Corticosteroids cause common and serious adverse effects due to their toxicity and/or withdrawal. Abrupt interruption of corticosteroids suppresses the endogenous cortisol by affecting the hypothalamic-pituitary-adrenal axis and leads to adrenal insufficiency. Main side effects of corticosteroids are fluid retention, hypertension, glucose intolerance/diabetes mellitus, osteoporosis, bone fractures, cataract, glaucoma, dyspepsia/gastrointestinal bleeding, loss of muscle mass/myopathy, skin thinning, bruising, striae rubrae, psychosis/depression, sleep disturbances, immunosuppression, infection risk, polycytemia, increased appetite and weight gain, adrenal suppression, and Cushing’s syndrome (Melani et al., 2023; Janahi et al., 2018; Chang et al., 2022; Edland and Waterer, 2025; Pirracchio et al., 2024).

In this special issue *“Safety of Corticosteroids in Respiratory Medicine,”* there are five articles on respiratory infections, lung cancer (LC) and sarcoidosis. The main points of these articles are highlighted in the following five paragraphs.

In their review on *“Corticosteroids in COVID-19: pros and cons,”* Bahsoun et al. have stated that corticosteroids reduce inflammation and mortality by downregulating cytokine storm and lung injury if given early in severe coronavirus disease-2019 (COVID-19) requiring oxygen treatment or mechanical ventilation. They have also emphasized that a prolonged higher-dose corticosteroid therapy is not superior to conventional/low dose therapy, causes adverse effects and reduces survival.

The retrospective study on *“The association between early corticosteroid use and the risk of secondary infections in hospitalized patients with COVID-19: a double-edged sword. Results from the international SCCM discovery viral infection and respiratory illness universal study (VIRUS) COVID-19 registry*” *by* Bansal et al. has aimed to investigate the impact of early corticosteroid use on the secondary infection rates within a large, multicenter and multinational data from 24 countries. Stratified by corticosteroid use within 48 h of admission, of the included 17,092 adult patients, overall 13.5% (corticosteroid group- 16.6% vs. non-corticosteroid group- 9.4%) has had one or more secondary bacterial infections (bacteremia, bacterial pneumonia, empyema, meningitis/encephalitis, septic shock, ventilator-associated pneumonia, secondary infection from unknown source) during hospitalization. While unadjusted univariate analysis has shown a higher risk for any secondary infection with corticosteroids (significant for bacteremia, bacterial pneumonia, septic shock and secondary infection from unknown source), after adjusting for confounding factors risks of any secondary infection, bacteremia, meningitis/encephalitis and secondary infection from unknown source has remained significantly high. The authors have concluded that corticosteroid use in hospitalized COVID-19 patients within 48 h of admission increases overall secondary infection risk with varying impacts on different infections: significant increase in bacteremia, weakening in bacterial pneumonia and septic shock, and a trend toward decrease in meningitis/encephalitis.

Another retrospective study on *“Association between the cumulative dose of glucocorticoids before the development of pneumonia and death in patients receiving long-term glucocorticoids: a secondary analysis based on a Chinese cohort study” by* Guo et al. has aimed to assess the association between the cumulative glucocorticoid dose and in-hospital case fatality in patients developing pneumonia while receiving long-term glucocorticoid treatment. The data from the included 625 patients has been obtained from the Dryad Digital Repository. The death rates in 30 and 90 days have been found to be 22.9% and 26.2%, respectively. After adjusting for confounders, the patients with different cumulative doses of glucocorticoids (1.5–2.95, 2.95–5, 5–11.5, and >11.5 g) have had lower risks for 30-day death compared with those in the lowest quintile (≤1.5 g). A statistically significant nonlinear, N-shaped association between the cumulative doses of glucocorticoids and 30-day death has been shown. This N-shaped association described as an increase in 30-day death with a cumulative dose up to 1.8 g, then a significant decrease up to 8 g, and an increase again above 8 g has been explained as follows. In the early stages of the treatment (up to 1.8 g), the case fatality increases with the challenging of the body by the underlying disease and sudden stimulation of glucocorticoids. In the second stage (up to 8 g), the underlying immune-related disease and its physical damage are improved and immune responses are suppressed; thus, the outcome is better even if pneumonia occurs. In the third stage (above 8 g), as tolerance to glucocorticoids and disease relapse might occur, pneumonia severity and case fatality can increase with increasing cumulative doses and duration of glucocorticoid treatment.

In their review *“Corticosteroids in lung cancer,”* Ćeriman Krstić et al. have expressed that in LC, corticosteroids are used for tumor-related disorders, therapeutic adverse effects, and/or symptoms. The authors have also noted that there is no clear evidence for their efficacy in LC although they are widely used for malignant airway obstruction, superior vena cava syndrome, brain metastases, treatment-related adverse events due to immunotherapy, chemotherapy or radiotherapy, anorexia and cachexia, fatigue, dyspnea, nausea/vomiting, spinal cord compression, and pain. Thus, they have recommended using corticosteroids at lower doses for a short time to provide safer management of LC.

In the narrative review *“Safety of corticosteroid therapy in sarcoidosis treatment,”* Di Marco Berardino et al. have emphasized the corticosteroid side effects reducing QoL in sarcoidosis although they remain the first-choice due to their rapid action. The authors recommend, as per the guidelines, starting with a dose of 5–10 mg/day, adjusting maintenance dose and duration by the efficacy/side effect ratio, and giving no corticosteroid treatment when there is no significant organ involvement and impairment in QoL assessed by validated methods.

In severe COVID-19 or bacterial CAP, moderate/severe Pneumocystis pneumonia in patients with human immunodeficiency virus infection, and septic shock and/or ARDS with infection, low-dose corticosteroids (≤400 mg hydrocortisone equivalent/day) for 7–10 days have been shown to decrease 28-/30-day or in-hospital mortality (Pirracchio et al., 2024). However, widespread corticosteroid use in CAP is not supported by published data as evidence shows it is more likely to cause harm than benefit. Blood glucose should be controlled tightly if they are used. Low-dose hydrocortisone may be given for 4–7 days in selected non-influenza cases with respiratory failure of less than 24 h and serum c-reactive protein above 200 mg/L (Edland and Waterer, 2025).

There are gray areas regarding indications/contraindications, dose and duration of corticosteroids in oncology owing to the absence of prospective and robust data. They can cause adverse events in LC during and/or after treatment (Faggiano et al., 2022). Corticosteroids used for LC-related symptoms may increase the risk of death and progression in non-small cell LC treated with immune checkpoint inhibitors but may be safe when used for treating immune-related adverse events, and may not affect immunotherapy-related survival benefits when used for non-LC-related symptoms. Thus, evaluating and individualizing indications rationally is required before administering corticosteroids in LC (Li et al., 2023).

No evidence supports corticosteroid use in IPF. In non-IPF progressive fibrotic ILD (hypersensitivity pneumonitis, connective tissue disease-related ILD), corticosteroid treatment is given first-line based on limited evidence. However, with predominating fibrotic component, corticosteroids will have no effect. In fibrotic ILD with inflammation, giving moderate-dose corticosteroids and re-evaluation 3 months later is recommended (Melani et al., 2023; Janahi et al., 2018; Funke-Chambour et al., 2024).

Treatment is not required in asymptomatic and non-progressive pulmonary sarcoidosis. In severe heart, lung, brain or renal involvement, and progressive symptomatic disease, low/moderate-dose corticosteroids are started, and maintenance dose and duration are adjusted to balance efficacy and side effects (Melani et al., 2023; Janahi et al., 2018).

In conclusion, from the editor’s viewpoint, systemic corticosteroid use in all respiratory diseases, including also COPD and asthma, should be prudent and limited. Clinically meaningful dose reductions are necessary if discontinuation is not possible. Cautious monitoring for and treating early side effects appropriately is a must. Studies with tighter entry criteria and better consideration of target groups, corticosteroid used, therapeutic window for benefit, and hyperglycemia will resolve disparate results.
